# Gamma-Aminobutyric Acid: A Novel Biomolecule to Improve Plant Resistance and Fruit Quality

**DOI:** 10.3390/plants14142162

**Published:** 2025-07-13

**Authors:** Jingrong Wang, Shaokun Sun, Wei Fang, Xin Fu, Fuguo Cao, Shujun Liu

**Affiliations:** 1 Liaoning Academy of Agricultural Sciences, Shenyang 110866, China; 18235431732@163.com (J.W.); sunshaokun2015@126.com (S.S.); 13610811112@163.com (W.F.); xinfushine@126.com (X.F.); 2 Liaoning Key Laboratory of Strawberry Breeding and Cultivation, College of Horticulture, Shenyang Agricultural University, Shenyang 110866, China

**Keywords:** γ-aminobutyric acid (GABA), transcriptional regulation, fruit ripening, abiotic stress

## Abstract

Gamma-aminobutyric acid (GABA), a ubiquitous non-protein amino acid, plays a vital role in the response of plants to biotic and abiotic stresses. This review summarizes the underlying mechanisms through which GABA contributes to plant stress resistance, including its biosynthetic and metabolic pathways, as well as its regulatory roles in enhancing stress tolerance and improving fruit quality. In plants, GABA is primarily synthesized from glutamate by the enzyme glutamate decarboxylase (GAD) and further metabolized by GABA transaminase (GABA-T) and succinic semialdehyde dehydrogenase (SSADH). The accumulation of GABA regulates various physiological and biochemical processes, including the control of stomatal closure, enhancement of antioxidant capacity, maintenance of ionic homeostasis, and stabilization of cellular pH. Moreover, GABA interacts with phytohormones to regulate plant growth, development, and stress tolerance. Notably, increasing GAD expression through genetic engineering has been shown to enhance tolerance to stresses, such as drought, saline-alkali, cold, and heat, in various plants, including tomato, rice, and creeping bentgrass. Additionally, GABA has effectively improved the storage quality of various fruits, including citrus fruits, apples, and strawberries. In conclusion, GABA holds significant research potential and promising applications in agricultural production and plant science.

## 1. Introduction

As sedentary organisms, plants are exposed to a wide range of environmental challenges throughout their growth and development [1]. Adverse environmental conditions significantly impact plant performance, and in recent years, the increasing frequency of extreme climate events—such as drought, high and low temperatures—has further intensified these stresses. Additionally, soil salinization, exacerbated by ongoing human activities, has become a major constraint on crop productivity. Together, these factors pose serious threats to agricultural sustainability and global food security [2,3,4].

While appropriate agronomic practices can help mitigate the effects of environmental stress, the application of exogenous regulatory compounds has also proven effective in enhancing plant resilience. Substances such as melatonin [5], jasmonic acid [6], strigolactones [7], γ-aminobutyric acid (GABA) [8], and salicylic acid [3] have been widely studied for their roles in modulating plant responses to abiotic stress. Among them, GABA, a four-carbon, non-protein amino acid, is widely distributed across all domains of life, including bacteria and eukaryotes [9,10]. GABA was first identified in potato tubers, and since then, a growing body of research has shown that GABA is widely distributed across various plant tissues and organs. It has been recognized as a metabolic substance involved in regulating pH, the C/N balance, plant development, and plant defense [11,12,13,14]. In recent years, several physiological and genetic studies suggest that GABA may also function as a signaling molecule that plays a role in regulating plant responses to abiotic stresses [15,16,17]. Given GABA’s potential in mitigating plant adversity and its value as a sustainable natural compound, it has received great attention from basic to applied research. However, the molecular mechanisms of GABA’s regulation of abiotic stress in plants are worthy of further investigation. In this review, we summarize the synthesis of GABA in plants and recent research advances on its role in response to plant growth, abiotic stress, and fruit quality, aiming to provide a theoretical foundation for a deeper understanding of GABA’s function in enhancing plant stress tolerance.

## 2. GABA Biosynthesis and Metabolism in Plants

GABA is mainly synthesized from glutamic acid (Glu) through an irreversible process catalyzed by glutamic acid decarboxylase (GAD). This process is conserved from bacteria to higher organisms [18,19]. Therefore, there is a direct relationship between glutamate levels and GABA levels. Plant GAD contains calmodulin (CaM)-binding domains, and its in vitro activity is optimal at pH 7.0–7.5 [14,20]. However, at acidic pH, GAD activity becomes less dependent on Ca^2+^-CaM and determines an optimal pH around 5.8 [20,21]. GAD can exist as either a dimer or a hexamer. Under physiological conditions, GAD mainly exists as a dimer with low activity. As the cytoplasmic pH decreases, GAD activity increases, with an optimal activity at pH 5.8. When the cytoplasmic pH falls below physiological levels, GAD loses its ability to bind to Ca^2+^-CaM [22]. This leads to the activation of GADase in a pH-dependent manner, a phenomenon that often occurs during acute stress and chronic stress [21]. In addition, *GAD* genes in plants usually exist as multigene families, and different *GAD* genes may be expressed in different tissues and developmental stages and may have different response patterns to different stresses [23]. For example, five *GAD* isoforms have been identified in *Arabidopsis thaliana*. Different GADs are expressed in a tissue-dependent manner. *AtGAD1* is mainly expressed in roots, while *AtGAD2* is expressed in all organs [24].

**Figure 1:**
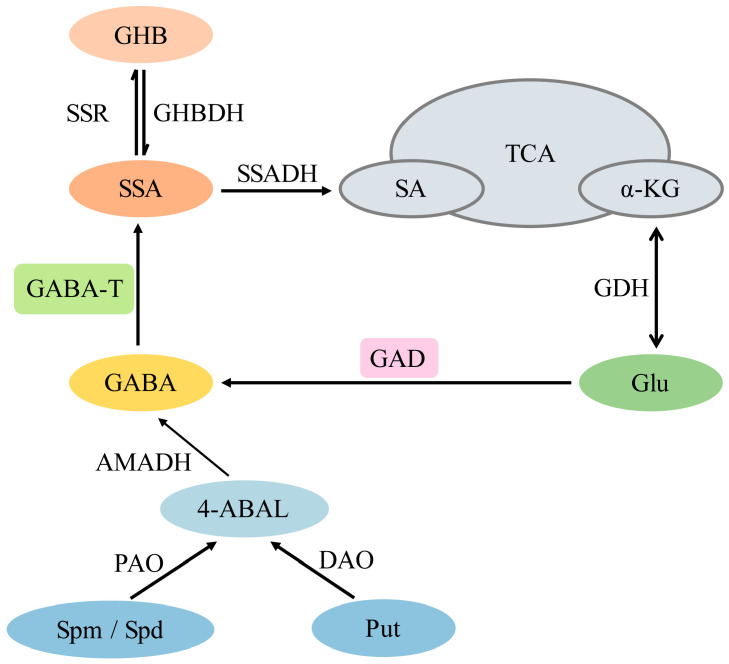
Synthesis and metabolic pathways of GABA in plants. Abbreviations: Glu, glutamate; GAD, glutamate decarboxylase; GABA-T, GABA transaminase; SSA, succinic semialdehyde; SSR, succinic semialdehyde reductase; GHB, gamma-hydroxybutyric acid; GHBDH, GHB dehydrogenase; SSADH, succinate semialdehyde dehydrogenase; SA, succinic acid; α-KG, α-ketoglutaric acid; GDH, glutamate dehydrogenase; Put, putrescine; Spm, spermine; Spd, spermidine; PAO, polyamineoxidase; DAO, diamine oxidase; 4-ABAL, 4-aminobutanal; AMADH, aminoaldehyde dehydrogenase.

Polyamine metabolism is another indirect source of GABA. In this pathway, spermidine is converted into putrescine through a multistep process. Spermidine is then converted to spermine. O_2_-dependent polyamine oxidase and diamine oxidase then catalyze the oxidation or degradation of spermine and putrescine to produce GABA and β-alanine, respectively (Figure 1) [25,26].

The degradation of GABA is mainly catalyzed by two enzymes: GABA transaminase (GABA-T) and succinate semialdehyde dehydrogenase (SSADH). GABA-T catalyzes the conversion of GABA to succinate semialdehyde (SSA), which is subsequently oxidized to succinate by SSADH, entering the tricarboxylic acid cycle. This pathway is known as the GABA bypass and plays an important role in plant metabolism and stress tolerance [14]. Alternatively, SSA can be reduced to γ-hydroxybutyrate (GHB) by succinate semialdehyde reductase (SSR) [27]. Studies have shown that GHB occurs naturally in trace amounts in the mammalian central nervous system [28]. Other experiments have confirmed that plants possess the ability to synthesize GHB in response to hypoxic stress, but its specific physiological function in plants has not yet been fully elucidated [29].

## 3. Role of GABA in Alleviating Abiotic and Biotic Stress

### 3.1. The Role of GABA in Enhancing Plant Tolerance to Temperature Stress

Cold stress is an important environmental factor that severely affects plant growth, development, and productivity. Increasing evidence has revealed that GABA is involved in the low-temperature regulatory mechanisms in plants. For instance, GABA levels are significantly elevated in barley seedlings exposed to cold stress, and the accumulation of GABA induces the expression of genes in the GABA shunt pathway, supporting the notion that GABA metabolism contributes to cold tolerance in plants [30].

This stress alters lipid composition and membrane fluidity, which can damage cellular structures and lead to the leakage of important solutes and ions [31]. Interestingly, GABA has been shown to protect cell membranes by regulating lipid composition, thereby stabilizing the plasma membrane and preventing oxidative damage [32].

**Figure 2:**
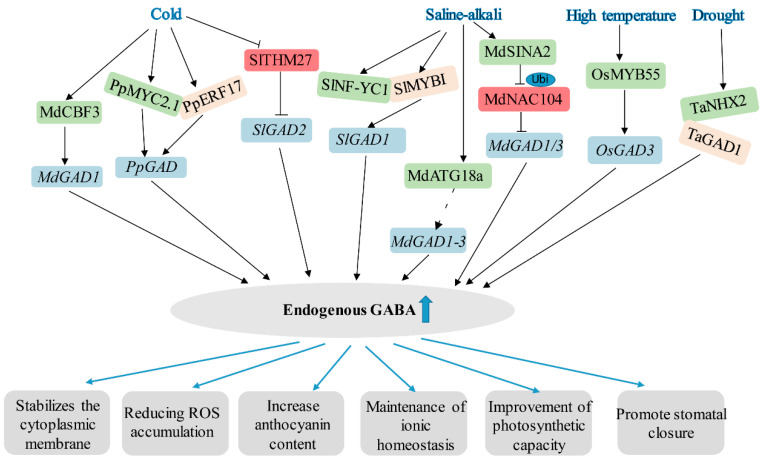
Transcriptional regulation of GADs activity in response to plant adversity. The thick blue arrow represents elevated endogenous GABA content. The black dashed arrow represents indirect regulation. The green boxes represent that the protein plays a positive regulatory role in this pathway. The beige box represents a protein that interacts with the protein in the green box. The red box represents that the protein plays a negative regulatory role in this pathway.

Additionally, low temperature stress leads to the accumulation of reactive oxygen species (ROS), which affect protein and DNA structures and damage biofilms and plant tissues, thereby inhibiting plant growth [10]. Under low-temperature stress, GABA levels and expression of GABA transporter-related genes increased in barley and wheat, while glutamate (the precursor of GABA) concentrations were significantly reduced. Thus, the biosynthesis of GABA to enhance cold tolerance may be associated with enhanced glutamate availability and GAD activity [33]. In another study, exogenous GABA improved physiological tolerance in various plants, such as wheat, tomato, and tea tree, by enhancing the antioxidant system and reducing oxidative damage associated with low temperature stress [10,30,34]. The production of GABA is regulated by a variety of factors, among which *GAD* has been identified as a key gene in the GABA synthesis pathway in a variety of plants. It was found that there are five genes (*GAD1–5*) in the *GAD* family in tomato, among which *SlGAD2* plays an important function under low temperature stress. The overexpression of *SlGAD2* increased endogenous GABA levels and anthocyanin content in transgenic tomato seedlings, thereby enhancing cold tolerance in tomato plants [10]. Similarly, the overexpression of *MdGAD1* in apple callus enhanced cold tolerance in apple by increasing the synthesis of endogenous GABA and enhancing the activity of antioxidant enzymes (Figure 2) [35].

Similarly, heat stress is a limiting environmental factor that negatively impacts plant growth. When plants are subjected to high-temperature stress, their physiological and biochemical systems undergo changes in response at multiple levels, including impaired structural and functional integrity of cell membranes, disruption of tissue water balance; significant alterations in the composition of products of primary metabolism (e.g., sugars, amino acids) and secondary metabolism (e.g., phenolics, terpenoids); and aberrations in protein conformation and lipid composition [36,37]. These chain reactions, from the molecular to the physiological level, ultimately interfere with the normal growth and developmental processes of the plant [36,38]. A variety of plant growth regulators are directly or indirectly involved in a wide range of physiological processes that confer heat tolerance to plants in response to high temperature stress. According to Li et al. (2016) [39], exogenously applied GABA effectively enhanced the heat tolerance of creeping bentgrass. This was achieved through improvements in antioxidant metabolism, inhibition of leaf senescence, regulation of photosynthesis and transpiration, and enhancement of osmoregulation, as well as the level of amino acids, carbohydrates, organic acids, and alcohols [39]. These findings suggest that GABA can systematically regulate primary metabolic processes in rice under high temperature stress, including multiple metabolic pathways such as amino acid, organic acid, and sugar metabolism, showing multi-target physiological regulation.

GABA-treated rice plants exhibited higher levels of both enzymatic and non-enzymatic antioxidants, which contributed to increased tolerance to high-temperature stress. This was achieved by maintaining leaf cell turgor pressure through enhanced osmolyte accumulation and reduced oxidative damage from antioxidant stimulation [40]. Additionally, GABA helped maintain relative water content in cells under high-temperature stress. For instance, Liu et al. (2019) [41] discovered that the chlorophyll content (Chl), Fv/Fm ratio, net photosynthesis (Pn), and water use efficiency (WUE) of GABA-pretreated stoloniferous grasses showed significantly higher values. This significantly enhanced the photosynthetic capacity of stoloniferous grasses under high-temperature stress, reducing water loss and maintaining water balance [41]. However, further studies are still needed to explain the potential molecular mechanisms by which GABA regulates heat stress tolerance.

### 3.2. The Role of GABA in Enhancing Plant Tolerance to Saline-Alkali Stress

Saline-alkali stress is usually characterized by high salinity and high pH [42]. Compared with neutral salt stress, the high pH associated with saline-alkali conditions not only intensifies the inhibitory effects of Na^+^ on the uptake of essential elements such as K^+^, Ca^2+^, and Mg^2+^, leading to the precipitation of metal ions in the rhizosphere and impaired nutrient absorption, but also induces a pronounced oxidative stress response. In combination with osmotic stress and ionic toxicity, these effects synergistically exacerbate cellular damage, making saline-alkali stress significantly more detrimental to plant growth than either salinity or alkalinity alone [43].

Ca^2+^ signaling has been reported to be the main response in plant cells under salinity stress. However, plants have evolved other physiological, molecular, and cellular adaptations to reduce their susceptibility to adverse conditions [44,45,46]. One such adaptation is the regulation of plant tolerance mechanisms by GABA. The overexpression of the *GAD* gene leads to a significant accumulation of GABA in tobacco leaves, which enhances its salt tolerance [47]. Xiang et al. (2016) suggested that exogenous GABA can function as an osmotic substrate that potentially reduces saline-alkali stress damage to mesophyll cell walls [48]. Exogenous application of GABA has been shown to enhance saline-alkali tolerance in various plants, including *Arabidopsis*, clover, apple, tomato, melon, rice, and sorghum. This effect is achieved by promoting chlorophyll synthesis, enhancing photosynthetic capacity, increasing antioxidant enzyme activity and AsA-GSH cycling, and decreasing Na^+^ accumulation and Na/K ratio to maintain ion homeostasis [8,49,50,51,52,53]. Zhang et al. (2025) reported that GABA treatment alleviated salt stress-induced physiological and metabolic damage in strawberry seedlings by enhancing the accumulation of osmolytes and polyamines and reducing oxidative damage [54].

Under salt stress, exogenous GABA increased the expression of SOS pathway-related genes (*SOS1*, *SOS2*, and *SOS3*), as well as ion transporter genes such as *HKT1* and *AKT1*, thereby promoting Na^+^ efflux and maintaining ion homeostasis, thus improving the salt tolerance in *C. triticina* [51]. In soybean, exogenous GABA reduces salinity-associated stress damage by decreasing the concentrations of the harmful substances such as Na^+^, Cl^−^, H_2_O_2_, and MDA, while increasing the content of photosynthetic pigments, mineral nutrients, and osmotic substances [55]. Additionally, there is a significant accumulation of glutamate (a GABA precursor) in the root systems of *gaba-t* mutant plants under salt stress. They also observed changes in cell wall composition, hypocotyl development, and the expression levels of sucrose-related genes and starch metabolism in the mutant plants. These findings confirm that GABA plays an important role in carbon adjustment under salt stress [56].

The adaptive responses of plants to environmental stresses are mediated through the interaction of multiple signaling pathways. These signaling networks (e.g., synergistic or antagonistic effects of factors such as phytohormones, active proteins, kinase cascades, and reactive oxygen species) work together to regulate the plant’s stress response [57,58]. Studies have shown that GABA regulates the expression of genes related to phytohormones in plants. For instance, GABA stimulates ethylene biosynthesis in sunflowers by promoting the expression of the ACC synthase gene [59]. The overexpression of *SlGAD1* increased the endogenous GABA content in transgenic tomato seedlings under saline-alkali stress, which also promoted ethylene accumulation, further enhancing the saline-alkali tolerance of tomato [8]. In *Cassia*, GABA-treated plants exhibited greater salt tolerance, which was closely associated with elevated levels of the growth hormone gibberellin (GA) and the regulation of other growth hormones, including indole-3-acetic acid (IAA) and indole-3-butyric acid (IBA) [60]. In addition, GABA has been found to interact with a variety of phytohormones such as ABA, salicylic acid, and jasmonic acid to help plants cope with abiotic stress [17,61,62]. However, the precise mechanisms by which GABA affects phytohormones remain controversial.

### 3.3. The Role of GABA in Enhancing Plant Tolerance to Drought Stress

In the context of changing global climatic conditions, drought stress is a major challenge for plants. It results from an insufficient water supply to the root system and elevated soil toxicity. These factors make plants more vulnerable to high levels of irradiation, which in turn reduces crop yield and quality [58,63]. Plants respond to drought stress through a variety of physiological and biochemical mechanisms, including the accumulation of osmoprotectants, activation of antioxidant systems, and adjustment of gene expression [63,64]. GABA is a key component of the plant drought response and plays an important role in improving plant drought tolerance. Under drought stress, exogenous application of GABA has been shown to alleviate leaf damage, as evidenced by increased relative water content and reductions in electrolyte leakage, lipid peroxidation, and leaf wilting rate [65]. Excessive production of ROS, a hallmark of drought-induced oxidative stress, is closely associated with cellular damage. The extent of ROS production can be influenced by several factors, including plant genotype, developmental stage, and the severity and duration of drought stress [66,67].

GABA treatment has been shown to significantly increased the activity of antioxidant enzymes, including superoxide dismutase (SOD), peroxidase (POD), catalase (CAT), ascorbate peroxidase (APX), and glutathione reductase (GR), while simultaneously reducing hydrogen peroxide (H_2_O_2_) production, thereby improving drought tolerance in sunflower [64]. A similar study on black pepper revealed that GABA alleviates drought stress by maintaining membrane stability, enhancing photosynthesis, and boosting CO_2_ uptake, proline content, and total sugar content [68,69]. Interestingly, genes encoding GABA receptors were highly expressed in drought-tolerant barley varieties under drought stress [70]. This is consistent with studies on the *gad1/2* mutant of *Arabidopsis* plants, which are more sensitive to drought and have lower GABA content [71]. These findings suggest that GABA plays an important role in plant resistance to drought stress. They also imply that GABA could potentially increase drought tolerance by regulating physiological and biochemical pathways.

The regulation of stomatal aperture plays a key role in plant productivity and drought tolerance, and has a profound impact on climate by influencing the global carbon and water cycles [17,72]. GABA plays a protective role against drought stress in plants by increasing osmolytes and leaf expansion and reducing oxidative damage through antioxidant regulation [63]. Cytoplasmic GABA produced by *GAD2* signaling reduces stomatal opening and transpiration water loss by negatively regulating ALMT9 activity, thereby improving water use efficiency and drought tolerance [17]. In wheat, TaNHX2 targets the C-terminal auto-inhibitory structural domain of TaGAD1, enhances its activity, and acts as a positive regulator in wheat drought resistance by promoting GABA accumulation and regulating stomata (Figure 2) [19].

In addition, GABA has been implicated in modulating polyamine (PA) metabolism, which is critical for plant stress tolerance [73]. Yong et al. (2017) demonstrated that exogenous GABA promotes PA biosynthesis while inhibiting PA catabolism, resulting in elevated levels of various PA types under drought stress, which in turn contributed to improved drought tolerance in white clover [65]. These results indicate the significance of GABA in plant adaptation to drought stress and imply that GABA may potentially manipulate physiological and biochemical pathways in plants to make them more resistant to drought stress.

### 3.4. The Role of GABA in Enhancing Plant Tolerance to Other Stresses

Under adverse conditions, the overproduction of ROS typically damages plant cellular components. However, growing evidence suggests that stress-induced ROS also play a signaling role [74]. For example, during wheat seed development, the reduction of hydrogen peroxide helps regulate the transcriptional activation of certain genes by establishing a specific redox environment [75]. In addition, ROS activate glutamate dehydrogenase (GDH) activity in mitochondria, promoting the production of glutamate, which then becomes an important raw material for the biosynthesis of ornithine and GABA [76].

It is believed that GABA accumulation helps reduce ROS-induced oxidative damage, thereby increasing tolerance to a wide range of stresses, such as hypoxia, high temperature, low light, and heavy metal stress [41,77,78,79]. Under hypoxia conditions, ROS (specifically H_2_O_2_) accumulate, leading to loss of cell viability. Interestingly, higher concentrations of GABA in the *pop2–5* mutant resulted in lower H_2_O_2_ levels compared to wild type or *gad1,2* plants. This may be due to the fact that elevated GABA restores membrane potential by regulating pH-dependent modulation of H^+^-ATPase activity and prevents excessive H_2_O_2_ accumulation by better controlling the ROS-Ca^2+^ center through transcriptional regulation of the *RBOH* gene [80]. Moreover, disturbances in membrane potential caused by hypoxic conditions significantly reduce intracellular carbohydrate content in plant cells. GABA plays a dual role in this process: it promotes sugar transport by optimizing the carbon-to-nitrogen ratio, and it maintains energy metabolism by modulating TCA cycle intermediates. Notably, the dual regulation of intracellular pH homeostasis and the TCA cycle by GABA is an important molecular mechanism through which plants resist hypoxia injury [58]. The overexpression of *OsMYB55* improved rice tolerance to high temperature by activating the expression of *OsGS1*, *GAT1*, and *GAD3*, which collectively promoted the accumulation of glutamate, GABA, and arginine (Figure 2) [81]. Exposure to UV light causes oxidative damage in *cam* mutant *Arabidopsis* plants. Under these conditions, the accumulation of GABA in the mutant plants is impaired, which reduces their tolerance to UV stress [69]. In addition, treatment of barley seedlings with exogenous GABA significantly alleviated the inhibitory effects of Al^3+^ and H^+^ on root growth. This positive effect of GABA was found to be due to its ability to reduce oxidative damage by strengthening the antioxidant defense system and decreasing carbonylated protein levels [82]. Furthermore, mustard seedlings treated with GABA exhibited tolerance to chromium (Cr) stress, as evidenced by reduced Cr uptake, increased relative water content and chlorophyll content in the leaves, and up-regulation of both enzymatic (APX, glutathione reductase, CAT, SOD, glyoxalase I, and glyoxalase II) and non-enzymatic (ascorbic acid and glutathione) antioxidants [83].

In addition, GABA also triggers a defense response in plants subjected to biotic stress, particularly insect attack, with a positive correlation between GABA levels and resistance to such stress [61]. Several studies have shown that elevated GABA levels in plants deter insect larvae from feeding. Additionally, the accumulation of GABA attenuates the toxic effects of certain compounds produced during insect feeding [84]. In another study, herbivore feeding on leaves led to a rapid increase in intracellular calcium ion levels in *Arabidopsis* within 1–2 min [85]. This finding may explain why GABA levels rise in unattacked leaves near injured tissues, supporting the idea that GABA plays a role in systemic defense against herbivorous insects. GABA also plays a crucial role in resistance to pathogens. For example, the inhibition of GABA biosynthesis-related genes, such as *GAD2* and *SSADH1*, reduced resistance to *Ralstonia solanacearum* in tomato [86]. Additionally, increasing GABA levels effectively reduced gray mold symptoms in tomato fruits [87]. In summary, the regulation of GABA metabolism is emerging as a key mechanism through which plants can effectively respond to various biological interactions.

## 4. The Role of GABA in Improving Fruit Quality

**Figure 3:**
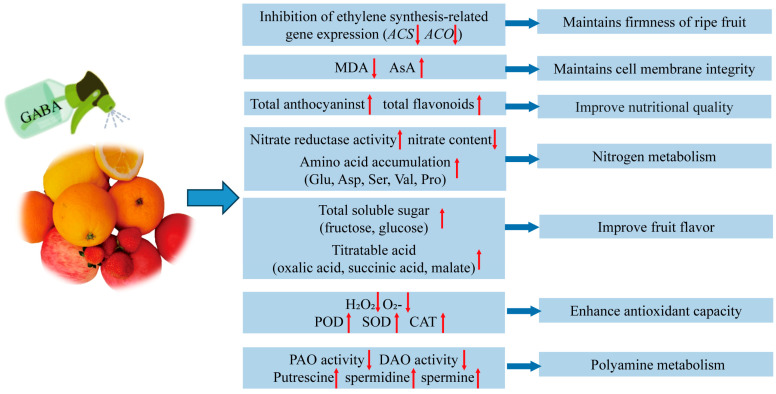
The Model of possible changes in fruit quality induced by GABA treatment. Glu, glutamic acid; Asp, aspartic acid; Ser, serine; Val, valine; Pro, proline; PAO, polyamine oxidase; DAO, diamine oxidase. The red up or down arrows represent an increase or decrease.

In addition to its significant effect in mitigating biotic and abiotic stress damage in plants, GABA also has a high potential in enhancing fruit quality (Figure 3). Studies have demonstrated that exogenous GABA treatment can effectively enhance the storage quality of citrus fruits. Specifically, GABA treatment significantly increased the levels of citric acid and various amino acids, including glutamic acid, alanine, serine, aspartic acid, and proline, thereby contributing to the improved flavor and nutritional value [88].

Studies have found that GABA inhibits fruit weight loss by maintaining cell membrane integrity and reducing respiration rates [89,90]. As such, Exogenous GABA reduced fruit weight loss while increasing total soluble sugar and titratable acid contents in strawberries. Moreover, treatment with 10 mM GABA significantly increased the activities of SOD and CAT, which helped to improve the antioxidant capacity of strawberries and thus prolonged their shelf life [89]. In apples, exogenous GABA increased the sugar-acid ratio in “Cipps Pink” fruits, increased flesh firmness and skin extensibility, and effectively improved fruit quality [91]. Additionally, GABA treatment helped maintain apple fruit quality by regulating ethylene synthesis, metabolism, polyamine metabolism, and the GABA shunt pathway [92]. In tomato, GABA metabolism is closely related to succinate accumulation during fruit ripening. GABA is rapidly converted into succinate by GABA transaminase (GABA-T) and succinate semialdehyde dehydrogenase (SSADH) activities, a process that is critical for fruit ripening and flavor development [93]. Similarly, in maize bean, exogenous GABA was shown to enhance both the activity and gene expression of enzymes involved in nitrogen metabolism and flavonoid biosynthesis, thereby significantly improving its nutritional quality [94]. GABA plays a positive role in maintaining the firmness of ripe fruits. For instance, Yan et al. (2024) discovered that 10 mM GABA delayed kiwifruit softening by preserving starch levels by suppressing the expression of genes that degrade starch (*AcGWD*, *AcPWD*, and *AcBAMs*) [95]. Furthermore, some studies have found that the application of exogenous GABA also reduced chilling injury in banana peel and peach fruits during cold storage [96,97,98]. In summary, GABA plays multiple roles in improving fruit quality, including increasing the nutritional value, enhancing flavor, extending shelf life, and boosting antioxidant capacity. These studies provide a theoretical foundation and practical guidance for the application of GABA in fruit storage and processing.

Interestingly, the researchers discovered that the accumulation of GABA was strongly associated with fruit dwarfism and sterility [93]. They also observed that high levels of GABA in leaves suppressed the expression of genes involved in cell elongation, thereby reducing the size of tomato plants [99]. In another study, higher levels of GABA in *SlGAD3*-overexpressing transgenic tomatoes resulted in reduced fruit sensitivity to ethylene [100]. These reports suggest that GABA metabolic homeostasis plays an important role in fruit development. Moreover, fluctuations in GABA content in plants may serve as a signaling mechanism. However, the exact mechanism by which plants recognize such changes remains unclear.

## 5. Conclusions

GABA is emerging as a crucial molecule in the quest to enhance plant resilience against the growing challenges posed by climate change. As a non-protein amino acid, GABA plays a multifaceted role in regulating plant stress responses, making it an invaluable tool for agricultural sustainability. Under stressful environmental conditions, such as drought, salinity, or temperature extremes, plants undergo various physiological disruptions. GABA mitigates these disruptions through several key mechanisms, including regulating stomatal closure to conserve water, boosting antioxidant defense systems to neutralize reactive oxygen species (ROS), and maintaining ion homeostasis, which helps stabilize cellular functions. Moreover, GABA’s ability to stabilize cellular pH ensures that enzymes and other vital molecules continue to function properly under stress, further protecting plant cells from damage. A particularly important feature of GABA is its interaction with various phytohormones, such as abscisic acid (ABA), auxins, and gibberellins, which coordinate to control plant growth, development, and responses to environmental stress. GABA not only aids in mitigating stress but also improves fruit quality by enhancing traits such as size, flavor, and shelf life.

Through genetic engineering to increase GABA levels, scientists have been able to enhance plant stress tolerance and improve crop productivity. This approach holds promise for developing crops that can withstand diverse abiotic stresses, thereby ensuring global food security in the face of unpredictable climate patterns.

## 6. Future Direction

Although current studies have revealed multiple mechanisms of action of GABA in plant stress tolerance and fruit quality improvement, some shortcomings remain. For example, the signaling pathways of GABA have not been fully elucidated, and the relationship between GABA and other metabolic pathways needs further investigation. Future research should aim to clarify the mechanisms of action of GABA receptors and their downstream signaling molecules and to uncover how GABA transmits stress signals within plant cells to trigger appropriate defense responses. Additionally, the specific role of GABA in plant resistance to pests and diseases warrants further exploration, with the goal of developing GABA-based biocontrol strategies.

Enhancing endogenous GABA levels through genetic engineering, metabolic engineering, or chemical induction holds great promise for breeding novel stress-resistant crop varieties that can better withstand the challenges of climate change. Through comprehensive and in-depth research, the precise application of GABA can be realized, thereby providing robust scientific support for ensuring food security and promoting sustainable agricultural development.

## References

[1] Zhang H., Zhu J., Gong Z., Zhu J.K. (2022). Abiotic stress responses in plants. Nat. Rev. Genet..

[2] Haider S., Raza A., Iqbal J., Shaukat M., Mahmood T. (2022). Analyzing the regulatory role of heat shock transcription factors in plant heat stress tolerance: A brief appraisal. Mol. Biol. Rep..

[3] Raza A., Salehi H., Rahman M.A., Zahid Z., Madadkar Haghjou M., Najafi-Kakavand S., Charagh S., Osman H.S., Albaqami M., Zhuang Y. (2022). Plant hormones and neurotransmitter interactions mediate antioxidant defenses under induced oxidative stress in plants. Front. Plant Sci..

[4] Xu J., Liu H., Zhou C., Wang J., Wang J., Han Y., Zheng N., Zhang M., Li X. (2024). The ubiquitin-proteasome system in the plant response to abiotic stress: Potential role in crop resilience improvement. Plant Sci..

[5] Ahmad I., Zhu G., Zhou G., Liu J., Younas M.U., Zhu Y. (2023). Melatonin role in plant growth and physiology under abiotic stress. Int. J. Mol. Sci..

[6] Elsisi M., Elshiekh M., Sabry N., Aziz M., Attia K., Islam F., Chen J., Abdelrahman M. (2024). The genetic orchestra of salicylic acid in plant resilience to climate change induced abiotic stress: Critical review. Stress Biol..

[7] Qi J., Mao Y., Cui J., Lu X., Xu J., Liu Y., Zhong H., Yu W., Li C. (2024). The role of strigolactones in resistance to environmental stress in plants. Physiol. Plant.

[8] Wang J., Zhang Y., Wang J., Ma F., Wang L., Zhan X., Li G., Hu S., Khan A., Dang H. (2024). Promoting gamma-aminobutyric acid accumulation to enhances saline-alkali tolerance in tomato. Plant Physiol..

[9] Fromm H. (2020). GABA signaling in plants: Targeting the missing pieces of the puzzle. J. Exp. Bot..

[10] Wang J., Zhang Y., Wang J., Khan A., Kang Z., Ma Y., Zhang J., Dang H., Li T., Hu X. (2024). *SlGAD2* is the target of SlTHM27, positively regulates cold tolerance by mediating anthocyanin biosynthesis in tomato. Hortic. Res..

[11] Bouché N., Fait A., Bouchez D., Moller S.G., Fromm H. (2003). Mitochondrial succinic-semialdehyde dehydrogenase of the γ-aminobutyrate shunt is required to restrict levels of reactive oxygen intermediates in plants. Proc. Natl. Acad. Sci. USA.

[12] Dent C.E., Stepka W., Steward F.C. (1947). Detection of the free amino-acids of plant cells by partition chromatography. Nature.

[13] Shelp B.J., Bown A.W., McLean M.D. (1999). Metabolism and functions of gamma-aminobutyric acid. Trends Plant Sci..

[14] Yuan D., Wu X., Gong B., Huo R., Zhao L., Li J., Lu G., Gao H. (2023). GABA metabolism, transport and their roles and mechanisms in the regulation of abiotic stress (hypoxia, salt, drought) resistance in plants. Metabolites.

[15] Kinnersley A.M., Turano F.J. (2000). Gamma aminobutyric acid (GABA) and plant responses to stress. Crit. Rev. Plant Sci..

[16] Ramesh S.A., Tyerman S.D., Gilliham M., Xu B. (2017). Gamma-aminobutyric acid (GABA) signalling in plants. Cell Mol. Life Sci..

[17] Xu B., Long Y., Feng X., Zhu X., Sai N., Chirkova L., Betts A., Herrmann J., Edwards E.J., Okamoto M. (2021). GABA signalling modulates stomatal opening to enhance plant water use efficiency and drought resilience. Nat. Commun..

[18] Podlesakova K., Ugena L., Spichal L., Dolezal K., De Diego N. (2019). Phytohormones and polyamines regulate plant stress responses by altering GABA pathway. New Biotechnol..

[19] Li J., Liu X., Chang S., Chu W., Lin J., Zhou H., Hu Z., Zhang M., Xin M., Yao Y. (2024). The potassium transporter TaNHX2 interacts with TaGAD1 to promote drought tolerance via modulating stomatal aperture in wheat. Sci. Adv..

[20] Kabała K., Janicka M. (2024). Relationship between the GABA Pathway and Signaling of Other Regulatory Molecules. Int. J. Mol. Sci..

[21] Guo Z., Gong J., Luo S., Zuo Y., Shen Y. (2023). Role of Gamma-Aminobutyric Acid in Plant Defense Response. Metabolites.

[22] Gut H., Dominici P., Pilati S., Astegno A., Petoukhov M.V., Svergun D.I., Grütter M.G., Capitani G. (2009). A common structural basis for pH- and calmodulin-mediated regulation in plant glutamate decarboxylase. J. Mol. Biol..

[23] Lee J.H., Kim Y.J., Jeong D.Y., Sathiyaraj G., Pulla R.K., Shim J.S., In J.G., Yang D.C. (2010). Isolation and characterization of a glutamate decarboxylase (*GAD*) gene and their differential expression in response to abiotic stresses from *Panax ginseng* C. A. Meyer. Mol. Biol. Rep..

[24] Bouche N., Fromm H. (2004). GABA in plants: Just a metabolite?. Trends Plant Sci..

[25] Xu M., Yang Q., Bai G., Li P., Yan J. (2022). Polyamine pathways interconnect with GABA metabolic processes to mediate the low-temperature response in plants. Front. Plant Sci..

[26] Li L., Dou N., Zhang H., Wu C. (2021). The versatile GABA in plants. Plant Signal. Behav..

[27] Ahmad S., Fariduddin Q. (2024). Deciphering the enigmatic role of gamma-aminobutyric acid (GABA) in plants: Synthesis, transport, regulation, signaling, and biological roles in interaction with growth regulators and abiotic stresses. Plant Physiol. Biochem..

[28] Andriamampandry C., Taleb O., Viry S., Muller C., Humbert J.P., Gobaille S., Aunis D., Maitre M. (2003). Cloning and characterization of a rat brain receptor that binds the endogenous neuromodulator γ-hydroxybutyrate. FASEB J..

[29] Breitkreuz K.E., Allan W.L., Van Cauwenberghe O.R., Jakobs C., Talibi D., André B., Shelp B.J. (2003). A novel gamma-hydroxybutyrate dehydrogenase: Identification and expression of an *Arabidopsis* cDNA and potential role under oxygen deficiency. J. Biol. Chem..

[30] Mazzucotelli E., Tartari A., Cattivelli L., Forlani G. (2006). Metabolism of gamma-aminobutyric acid during cold acclimation and freezing and its relationship to frost tolerance in barley and wheat. J. Exp. Bot..

[31] Gatti L.V., Basso L.S., Miller J.B., Gloor M., Gatti Domingues L., Cassol H.L.G., Tejada G., Aragão L., Nobre C., Peters W. (2021). Amazonia as a carbon source linked to deforestation and climate change. Nature.

[32] Yu Y., Li M., Li C., Niu M., Dong H., Zhao S., Jia C., Xu Y. (2023). Accelerated accumulation of γ-Aminobutyric acid and modifications on its metabolic pathways in black rice grains by germination under cold Stress. Foods.

[33] Guy C., Kaplan F., Kopka J., Selbig J., Hincha D.K. (2008). Metabolomics of temperature stress. Physiol. Plant.

[34] Zhu X., Liao J., Xia X., Xiong F., Li Y., Shen J., Wen B., Ma Y., Wang Y., Fang W. (2019). Physiological and iTRAQ-based proteomic analyses reveal the function of exogenous gamma-aminobutyric acid (GABA) in improving tea plant (*Camellia sinensis* L.) tolerance at cold temperature. BMC Plant Biol..

[35] Liu T., Li Y., Shi Y., Ma J., Peng Y., Tian X., Zhang N., Ma F., Li C. (2024). γ-Aminobutyric acid mediated by MdCBF3- *MdGAD1* mitigates low temperature damage in apple. Int. J. Biol. Macromol..

[36] Sharma L., Priya M., Kaushal N., Bhandhari K., Chaudhary S., Dhankher O.P., Prasad P.V.V., Siddique K.H.M., Nayyar H. (2020). Plant growth-regulating molecules as thermoprotectants: Functional relevance and prospects for improving heat tolerance in food crops. J. Exp. Bot..

[37] Fragkostefanakis S., Röth S., Schleiff E., Scharf K.D. (2015). Prospects of engineering thermotolerance in crops through modulation of heat stress transcription factor and heat shock protein networks. Plant Cell Environ..

[38] Wang X., Hou L., Lu Y., Wu B., Gong X., Liu M., Wang J., Sun Q., Vierling E., Xu S. (2018). Metabolic adaptation of wheat grain contributes to a stable filling rate under heat stress. J. Exp. Bot..

[39] Li Z., Yu J., Peng Y., Huang B. (2016). Metabolic pathways regulated by γ-aminobutyric acid (GABA) contributing to heat tolerance in creeping bentgrass (*Agrostis stolonifera*). Sci. Rep..

[40] Nayyar H., Kaur R., Kaur S., Singh R. (2014). γ-Aminobutyric acid (GABA) imparts partial protection from heat stress injury to rice seedlings by improving leaf turgor and upregulating osmoprotectants and antioxidants. J. Plant Growth Regul..

[41] Liu T., Liu Z., Li Z., Peng Y., Zhang X., Ma X., Huang L., Liu W., Nie G., He L. (2019). Regulation of heat shock factor pathways by γ-aminobutyric acid (GABA) associated with thermotolerance of creeping bentgrass. Int. J. Mol. Sci..

[42] Cao Y., Song H., Zhang L. (2022). New insight into plant saline-alkali tolerance mechanisms and application to breeding. Int. J. Mol. Sci..

[43] Zhang K.Y., Chang L., Li G.H., Li Y.F. (2023). Advances and future research in ecological stoichiometry under saline-alkali stress. Environ. Sci. Pollut. Res..

[44] Köster P., Wallrad L., Edel K.H., Faisal M., Alatar A.A., Kudla J. (2019). The battle of two ions: Ca^2+^ signalling against Na^+^ stress. Plant Biol..

[45] Manishankar P., Wang N., Köster P., Alatar A.A., Kudla J. (2018). Calcium signaling during salt stress and in the regulation of ion homeostasis. J. Exp. Bot..

[46] Aliniaeifard S., Hajilou J., Tabatabaei S.J., Sifi-Kalhor M. (2016). Effects of ascorbic acid and reduced glutathione on the alleviation of salinity stress in olive plants. Int. J. Fruit. Sci..

[47] Akcay N., Bor M., Karabudak T., Ozdemir F., Turkan I. (2012). Contribution of gamma amino butyric acid (GABA) to salt stress responses of *Nicotiana sylvestris* CMSII mutant and wild type plants. J. Plant Physiol..

[48] Xiang L., Hu L., Xu W., Zhen A., Zhang L., Hu X. (2016). Exogenous γ-aminobutyric acid improves the structure and function of photosystem II in muskmelon seedlings exposed to salinity-alkalinity stress. PLoS ONE.

[49] Cheng B., Li Z., Liang L., Cao Y., Zeng W., Zhang X., Ma X., Huang L., Nie G., Liu W. (2018). The γ-aminobutyric acid (GABA) alleviates salt stress damage during seeds germination of white clover associated with Na⁺/K⁺ transportation, dehydrins accumulation, and stress-related genes expression in white clover. Int. J. Mol. Sci..

[50] Dabravolski S.A., Isayenkov S.V. (2023). The role of the γ-aminobutyric acid (GABA) in plant salt stress tolerance. Horticulturae.

[51] Shi Y., Li Y., Liu T., Guo C., Liang W., Ma F., Li C. (2024). Gamma-aminobutyric acid enhances salt tolerance by sustaining ion homeostasis in apples. Plant Physiol. Biochem..

[52] Su N., Wu Q., Chen J., Shabala L., Mithofer A., Wang H., Qu M., Yu M., Cui J., Shabala S. (2019). GABA operates upstream of H^+^-ATPase and improves salinity tolerance in *Arabidopsis* by enabling cytosolic K^+^ retention and Na^+^ exclusion. J. Exp. Bot..

[53] Yang Y., Guo Y. (2018). Elucidating the molecular mechanisms mediating plant salt-stress responses. New Phytol..

[54] Zhang Y., Deng M., Lin B., Tian S., Chen Y., Huang S., Lin Y., Li M., He W., Wang Y. (2025). Physiological and transcriptomic evidence revealed the role of exogenous GABA in enhancing salt tolerance in strawberry seedlings. BMC Genom..

[55] Zhao Q., Liu L., Wei Z., Bai Q., Zhao C., Zhang S., Pan J., Yu J., Zhang S., Wei J. (2024). Gamma-aminobutyric acid (GABA) improves salinity stress tolerance in soybean seedlings by modulating their mineral nutrition, osmolyte contents, and ascorbate-glutathione cycle. BMC Plant Biol..

[56] Renault H., El Amrani A., Berger A., Mouille G., Soubigou-Taconnat L., Bouchereau A., Deleu C. (2013). Gamma-aminobutyric acid transaminase deficiency impairs central carbon metabolism and leads to cell wall defects during salt stress in *Arabidopsis* roots. Plant Cell Environ..

[57] Rejeb I.B., Pastor V., Mauch-Mani B. (2014). Plant responses to simultaneous biotic and abiotic stress: Molecular mechanisms. Plants.

[58] Seifikalhor M., Aliniaeifard S., Hassani B., Niknam V., Lastochkina O. (2019). Diverse role of gamma-aminobutyric acid in dynamic plant cell responses. Plant Cell Rep..

[59] Kathiresan A., Tung P., Chinnappa C.C., Reid D.M. (1997). gamma-Aminobutyric acid stimulates ethylene biosynthesis in sunflower. Plant Physiol..

[60] Alqarawi A.A., Hashem A., Abd_Allah E.F., Al-Huqail A.A., Alshahrani T.S., Alshalawi S.a.R., Egamberdieva D. (2016). Protective role of gamma amminobutyric acid on *Cassia italica* Mill under salt stress. Legume Res.-Int. J..

[61] Hu Y., Huang X., Xiao Q., Wu X., Tian Q., Ma W., Shoaib N., Liu Y., Zhao H., Feng Z. (2024). Advances in plant GABA research: Biological functions, synthesis mechanisms and regulatory pathways. Plants.

[62] Kobayashi Y., Kobayashi Y., Sugimoto M., Lakshmanan V., Iuchi S., Kobayashi M., Bais H.P., Koyama H. (2013). Characterization of the complex regulation of *AtALMT1* expression in response to phytohormones and other inducers. Plant Physiol..

[63] Hasan M.M., Alabdallah N.M., Alharbi B.M., Waseem M., Yao G., Liu X.D., Abd El-Gawad H.G., El-Yazied A.A., Ibrahim M.F.M., Jahan M.S. (2021). GABA: A key player in drought stress resistance in plants. Int. J. Mol. Sci..

[64] Abdel Razik E.S., Alharbi B.M., Pirzadah T.B., Alnusairi G.S.H., Soliman M.H., Hakeem K.R. (2021). Gamma-aminobutyric acid (GABA) mitigates drought and heat stress in sunflower (*Helianthus annuus* L.) by regulating its physiological, biochemical and molecular pathways. Physiol. Plant.

[65] Yong B., Xie H., Li Z., Li Y.P., Zhang Y., Nie G., Zhang X.Q., Ma X., Huang L.K., Yan Y.H. (2017). Exogenous application of GABA improves PEG-induced drought tolerance positively associated with GABA-shunt, polyamines, and proline metabolism in white clover. Front. Physiol..

[66] Guler N.S., Pehlivan N. (2016). Exogenous low-dose hydrogen peroxide enhances drought tolerance of soybean (*Glycine max* L.) through inducing antioxidant system. Acta Biol. Hung..

[67] Hasan M.M., Skalicky M., Jahan M.S., Hossain M.N., Anwar Z., Nie Z.F., Alabdallah N.M., Brestic M., Hejnak V., Fang X.W. (2021). Spermine: Its emerging role in regulating drought stress responses in plants. Cells.

[68] Vijayakumari K., Puthur J.T. (2016). γ-Aminobutyric acid (GABA) priming enhances the osmotic stress tolerance in *Piper nigrum* Linn. plants subjected to PEG-induced stress. Plant Growth Regul..

[69] Al-Quraan N. (2015). GABA shunt deficiencies and accumulation of reactive oxygen species under UV treatments: Insight from *Arabidopsis* thaliana calmodulin mutants. Acta Physiol. Plant..

[70] Guo P., Baum M., Grando S., Ceccarelli S., Bai G., Li R., von Korff M., Varshney R.K., Graner A., Valkoun J. (2009). Differentially expressed genes between drought-tolerant and drought-sensitive barley genotypes in response to drought stress during the reproductive stage. J. Exp. Bot..

[71] Mekonnen D.W., Flügge U.-I., Ludewig F. (2016). Gamma-aminobutyric acid depletion affects stomata closure and drought tolerance of *Arabidopsis* thaliana. Plant Sci..

[72] Keenan T.F., Hollinger D.Y., Bohrer G., Dragoni D., Munger J.W., Schmid H.P., Richardson A.D. (2013). Increase in forest water-use efficiency as atmospheric carbon dioxide concentrations rise. Nature.

[73] Hu X., Xu Z., Xu W., Li J., Zhao N., Zhou Y. (2015). Application of γ-aminobutyric acid demonstrates a protective role of polyamine and GABA metabolism in muskmelon seedlings under Ca(NO_3_)_2_ stress. Plant Physiol. Biochem..

[74] Mittler R., Vanderauwera S., Gollery M., Van Breusegem F. (2004). Reactive oxygen gene network of plants. Trends Plant Sci..

[75] Pulido P., Dominguez F., Cejudo F.J. (2009). A hydrogen peroxide detoxification system in the nucleus of wheat seed cells: Protection or signaling role?. Plant Signal. Behav..

[76] Skopelitis D.S., Paranychianakis N.V., Paschalidis K.A., Pliakonis E.D., Delis I.D., Yakoumakis D.I., Kouvarakis A., Papadakis A.K., Stephanou E.G., Roubelakis-Angelakis K.A. (2006). Abiotic stress generates ROS that signal expression of anionic glutamate dehydrogenases to form glutamate for proline synthesis in tobacco and grapevine. Plant Cell.

[77] Yang R., Guo Y., Wang S., Gu Z. (2015). Ca^2+^ and aminoguanidine on γ-aminobutyric acid accumulation in germinating soybean under hypoxia-NaCl stress. J. Food Drug Anal..

[78] Li Y., Fan Y., Ma Y., Zhang Z., Yue H., Wang L., Li J., Jiao Y. (2017). Effects of exogenous γ-aminobutyric acid (GABA) on photosynthesis and antioxidant system in pepper (*Capsicum annuum* L.) seedlings under low light stress. J. Plant Growth Regul..

[79] Suhel M., Husain T., Prasad S.M., Singh V.P. (2023). GABA requires nitric oxide for alleviating arsenate stress in tomato and brinjal seedlings. J. Plant Growth Regul..

[80] Wu Q., Su N.N., Huang X., Cui J., Shabala L., Zhou M.X., Yu M., Shabala S. (2021). Hypoxia-induced increase in GABA content is essential for restoration of membrane potential and preventing ROS-induced disturbance to ion homeostasis. Plant Commun..

[81] El-Kereamy A., Bi Y.M., Ranathunge K., Beatty P.H., Good A.G., Rothstein S.J. (2012). The rice R2R3-MYB transcription factor OsMYB55 is involved in the tolerance to high temperature and modulates amino acid metabolism. PLoS ONE.

[82] Song H., Xu X., Wang H., Wang H., Tao Y. (2010). Exogenous gamma-aminobutyric acid alleviates oxidative damage caused by aluminium and proton stresses on barley seedlings. J. Sci. Food Agric..

[83] Mahmud J.A., Hasanuzzaman M., Nahar K., Rahman A., Hossain M.S., Fujita M. (2017). gamma-aminobutyric acid (GABA) confers chromium stress tolerance in *Brassica juncea* L. by modulating the antioxidant defense and glyoxalase systems. Ecotoxicology.

[84] Mirabella R., Rauwerda H., Struys E.A., Jakobs C., Triantaphylidès C., Haring M.A., Schuurink R.C. (2008). The *Arabidopsis her1* mutant implicates GABA in E-2-hexenal responsiveness. Plant J..

[85] Kiep V., Vadassery J., Lattke J., Maaß J.-P., Boland W., Peiter E., Mithöfer A. (2015). Systemic cytosolic Ca^2+^ elevation is activated upon wounding and herbivory in *Arabidopsis*. New Phytol..

[86] Wang G., Kong J., Cui D., Zhao H., Niu Y., Xu M., Jiang G., Zhao Y., Wang W. (2019). Resistance against *Ralstonia solanacearum* in tomato depends on the methionine cycle and the γ-aminobutyric acid metabolic pathway. Plant J..

[87] Sun C., Jin L., Cai Y., Huang Y., Zheng X., Yu T. (2019). L-Glutamate treatment enhances disease resistance of tomato fruit by inducing the expression of glutamate receptors and the accumulation of amino acids. Food Chem..

[88] Sheng L., Shen D., Luo Y., Sun X., Wang J., Luo T., Zeng Y., Xu J., Deng X., Cheng Y. (2017). Exogenous gamma-aminobutyric acid treatment affects citrate and amino acid accumulation to improve fruit quality and storage performance of postharvest citrus fruit. Food Chem..

[89] Zhang Y., Lin B., Tang G., Chen Y., Deng M., Lin Y., Li M., He W., Wang Y., Zhang Y. (2024). Application of gamma-aminobutyric acid improves the postharvest marketability of strawberry by maintaining fruit quality and enhancing antioxidant system. Food Chem. X.

[90] Saeedi M., Mirdehghan S.H., Nazoori F., Esmaeilizadeh M., Saba M.K. (2022). Impact of calcium and γ-aminobutyric acid (GABA) on qualitative attributes and shelf life characteristics of fresh in-hull pistachio during cold storage. Postharvest Biol. Technol..

[91] Cheng P., Yue Q., Zhang Y., Zhao S., Khan A., Yang X., He J., Wang S., Shen W., Qian Q. (2023). Application of gamma-aminobutyric acid (GABA) improves fruit quality and rootstock drought tolerance in apple. J. Plant Physiol..

[92] Li C., Zhu J., Sun L., Cheng Y., Hou J., Fan Y., Ge Y. (2021). Exogenous γ-aminobutyric acid maintains fruit quality of apples through regulation of ethylene anabolism and polyamine metabolism. Plant Physiol. Biochem..

[93] Koike S., Matsukura C., Takayama M., Asamizu E., Ezura H. (2013). Suppression of γ-aminobutyric acid (GABA) transaminases induces prominent GABA accumulation, dwarfism and infertility in the tomato (*Solanum lycopersicum* L.). Plant Cell Physiol..

[94] Yu G.B., Chen F.Q., Wang Y.T., Chen Q.S., Liu H.L., Tian J., Wang M.X., Ren C.Y., Zhao Q., Yang F.J. (2022). Exogenous γ-aminobutyric acid strengthens phenylpropanoid and nitrogen metabolism to enhance the contents of flavonoids, amino acids, and the derivatives in edamame. Food Chem. X.

[95] Yan W., Cao M., Shi L., Wu W., Xu F., Chen W., Yang Z. (2024). γ-Aminobutyric acid delays fruit softening in postharvest kiwifruit by inhibiting starch and cell wall degradation. Postharvest Biol. Technol..

[96] Shang H., Cao S., Yang Z., Cai Y., Zheng Y. (2011). Effect of exogenous γ-aminobutyric acid treatment on proline accumulation and chilling injury in peach fruit after long-term cold storage. J. Agric. Food Chem..

[97] Wang Y., Luo Z., Huang X., Yang K., Gao S., Du R. (2014). Effect of exogenous γ-aminobutyric acid (GABA) treatment on chilling injury and antioxidant capacity in banana peel. Sci. Hortic..

[98] Liu Q., Li X., Jin S., Dong W., Zhang Y., Chen W., Shi L., Cao S., Yang Z. (2023). γ-Aminobutyric acid treatment induced chilling tolerance in postharvest kiwifruit (*Actinidia chinensis* cv. Hongyang) via regulating ascorbic acid metabolism. Food Chem..

[99] Nonaka S., Arai C., Takayama M., Matsukura C., Ezura H. (2017). Efficient increase of ɣ-aminobutyric acid (GABA) content in tomato fruits by targeted mutagenesis. Sci. Rep..

[100] Takayama M., Matsukura C., Ariizumi T., Ezura H. (2017). Activating glutamate decarboxylase activity by removing the autoinhibitory domain leads to hyper γ-aminobutyric acid (GABA) accumulation in tomato fruit. Plant Cell Rep..

